# Ambulatory Toxicity Management (AToM) in patients receiving adjuvant or neo-adjuvant chemotherapy for early stage breast cancer - a pragmatic cluster randomized trial protocol

**DOI:** 10.1186/s12885-019-6099-x

**Published:** 2019-09-05

**Authors:** Monika K. Krzyzanowska, Jim A. Julian, Melanie Powis, Doris Howell, Craig C. Earle, Katherine A. Enright, Nicole Mittmann, Maureen E. Trudeau, Eva Grunfeld

**Affiliations:** 1 Princess Margaret Cancer Centre, University Health Network, Toronto, ON Canada; 2 Ontario Clinical Oncology Group, McMaster University, Hamilton, ON Canada; 3 Ontario Institute for Cancer Research, Toronto, ON Canada; 4 Trillium Health Partners, Credit Valley Hospital, Mississauga, ON Canada; 5 Cancer Care Ontario, Toronto, ON Canada; 6 Sunnybrook Health Sciences Centres, Toronto, ON Canada; 7 Department of Family and Community Medicine, University of Toronto, Toronto, ON Canada; 8 Department of Medicine, University of Toronto, Toronto, ON Canada

**Keywords:** Breast cancer, Symptom management, Chemotherapy toxicity, Telephone case management, Quality improvement

## Abstract

**Background:**

Population-based studies suggest that emergency department visits and hospitalizations are common among patients receiving chemotherapy and that rates in routine practice are higher than expected from clinical trials. Chemotherapy-related toxicities are often predictable and, consequently, acute care visits may be preventable with adequate treatment planning and support between visits to the cancer centre. We will evaluate the impact of proactive telephone-based toxicity management on emergency department visits and hospitalizations in women with early stage breast cancer receiving chemotherapy.

**Methods:**

In this pragmatic covariate constraint-based cluster randomized trial, 20 centres in Ontario, Canada are randomly allocated to either proactive telephone toxicity management (intervention) or routine care (control). The primary outcome is the cluster-level mean number of ED + H visits per patient evaluated using Ontario administrative healthcare data. Participants are all patients with early stage (I-III) breast cancer commencing adjuvant or neo-adjuvant chemotherapy at participating institutions during the intervention period. At least 25 patients at each centre participate in a patient reported outcomes sub-study involving the collection of standardized questionnaires to measure: severity of treatment toxicities, self-care, self-efficacy, quality of life, and coordination of care. Patients participating in the patient reported outcomes (PRO) sub-study are asked to provide written consent to link their PRO data to administrative data. Unit costs will be applied to each per person resource utilized, and a total cost per population and patient will be generated. An incremental cost-effectiveness analysis will be undertaken to compare the incremental costs and outcomes between the intervention and control groups from the health system perspective.

**Discussion:**

This study evaluates the effectiveness of a proactive toxicity management intervention in a routine care setting. The use of administrative healthcare data to evaluate the primary outcome enables an evaluation in a real world setting and at a much larger scale than previous studies.

**Trial registration:**

Clinicaltrials.gov, NCT02485678. Registered 30 June 2015.

**Electronic supplementary material:**

The online version of this article (10.1186/s12885-019-6099-x) contains supplementary material, which is available to authorized users.

## Background

The majority of contemporary chemotherapy is administered in outpatient settings and, as such, patients who experience toxicity do so at home between visits to the cancer clinic. Population-based studies suggest that emergency department visits and hospitalizations (ED + H) are common during chemotherapy for many malignancies including breast, lung and colon cancer, and lymphoma [1–6]. Many chemotherapy-related toxicities are predictable and, consequently, some ED + H visits may be preventable with adequate treatment planning and/or enhanced support between clinic visits [4, 7–11].

Proactive approaches to managing toxicity such as telephone based support or electronic symptom monitoring are emerging as strategies to decrease the need for acute care and improve outcomes in patients receiving chemotherapy in oncology [7–13]. While the existing studies have been shown to improve symptom control and quality of life, and, in some cases, to decrease ED visits, the majority of prior studies were conducted in the setting of metastatic cancer [12], or are either of small scale [13] or single institution studies [12]. Whether these promising findings will translate into improved patient or healthcare system outcomes upon larger scale implementation is not yet known. In our own jurisdiction, Ontario, Canada, a recent environmental scan of regional cancer centres performed by the provincial cancer agency found that few centres had proactive approaches to symptom management and that availability of care outside regular clinic hours including for telephone-based advice to manage acute problems was limited. These gaps along with local population-level data showing high rates of acute care utilization during chemotherapy for breast and colon cancer and lymphoma in Ontario [1, 2] were the impetus for developing and testing a new model for remote symptom assessment and management for patients undergoing chemotherapy.

A two-centre pilot study of proactive telephone-based symptom management in early stage breast cancer patients receiving adjuvant or neo-adjuvant chemotherapy demonstrated the feasibility and acceptability of delivering telephone symptom management in our target population (AToM Pilot) and was associated with a lower rate of ED + H compared to controls [13]. We now plan to conduct a large scale evaluation of this proactive telephone symptom management intervention informed by lessons learned in the pilot study. Herein we describe the protocol (version: 3.0; 12 December 2016) for a pragmatic, cluster randomized controlled trial (cRCT) to evaluate the effect of proactive, nurse-led telephone-based symptom management on cluster-level number of ED + H visits in patients receiving adjuvant or neo-adjuvant chemotherapy for early stage breast cancer using healthcare administrative data. We have targeted this population as breast cancer is a leading cause of morbidity and mortality in women worldwide [14, 15], and many women diagnosed with breast cancer require systemic treatment in the adjuvant or metastatic setting [16–19] which has been associated with high rates of acute care utilization [1, 2, 6].

## Methods

### Study design overview

**Fig. 1:**
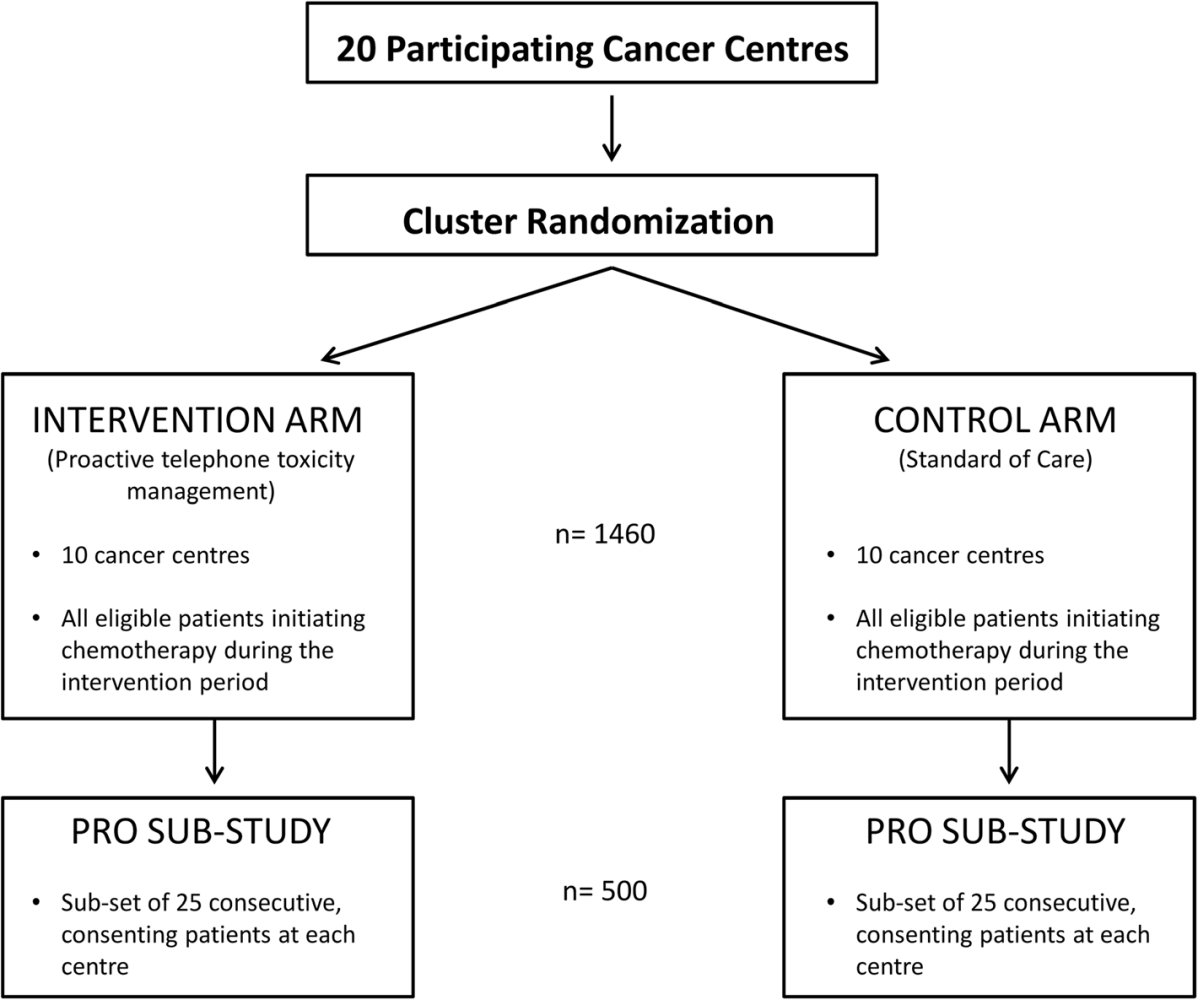
Schema for main study and patient reported outcomes (PRO) sub-study: A Pragmatic Cluster Randomized Trial of Ambulatory Toxicity Management in Patients Receiving Adjuvant or Neo-adjuvant Chemotherapy for Early Stage Breast Cancer (AToM)

The pragmatic covariate constraint-based cRCT involves 20 centres in Ontario, Canada that provide care to women with breast cancer (Fig. 1). Centres are randomly allocated to either proactive telephone symptom management (intervention) or routine care (control). Information regarding baseline supportive care, chemotherapy prescribing and follow-up practices is collected from all participating centres prior to study initiation using a Centre Baseline Survey. Participants include all patients with early stage (I-III) breast cancer commencing adjuvant or neo-adjuvant chemotherapy at participating institutions during the intervention period. The primary outcome is the cluster-level mean number of ED + H visits per patient and will be evaluated using Ontario administrative healthcare data (Table 1). Ontario has a single-payer universal healthcare system with a comprehensive population-based cancer registry capturing diagnostic and demographic information on approximately 98% of incident cancer cases [20]. Secondary outcomes will assess the effect of the intervention on: (1) severity of chemotherapy treatment toxicities; (2) self-care for management of chemotherapy toxicities; (3) self-efficacy (confidence) for managing symptoms (4) quality of life (including anxiety and depression); and (5) co-ordination of care. Unit costs will be applied to each per person resource utilized, and a total cost per population and patient will be generated. An incremental cost effectiveness analysis will be undertaken to compare the incremental costs and outcomes between the intervention and control groups from the health system perspective. A sub-set of at least 25 patients at each institution participate in the patient reported outcomes (PRO) sub-study which involves completion of standardized questionnaires. Patients participating in the PRO sub-study are asked to provide written consent to link their PRO data to administrative data. The study has been approved by the Ontario Cancer Research Ethics Board, a centralized ethics board used by 18 of the participating cancer centres, the Sault Area Hospital Research Ethics Board, and the Rouge Valley Health System Research Ethics Board (version 3; 12 Dec 2016).

**Table 1:** Summary of primary and secondary outcomes

Type	Outcome	Measurement
Primary Outcome	Cluster-level mean number of ED + H visits per patient	Counts extracted from healthcare administrative data
Patient Reported Outcomes (Secondary Outcomes)	Severity of chemotherapy treatment toxicities	National Cancer Institute Patient-reported Outcomes Common Terminology Criteria for Adverse Events (NCI-PRO-CTCAE)
	Self-care for management of chemotherapy toxicities	Leuven Questionnaire for Patient Self-Care during Chemotherapy (L-PaSC)
	Self-efficacy (confidence) for managing symptoms	Stanford Self-Management Self-Efficacy
	Quality of life (including anxiety and depression)	− Patient Health Questionnaire (PHQ-9) − Generalized Anxiety Disorder (GAD-7) − Functional Assessment for Cancer Therapy for Patients with Breast Cancer (FACT-B) − EQ-5D-3 L
	Coordination of care	Adapted Picker Survey
	Cost-effectiveness of pro-active toxicity management compared to routine care (control arm)	− Healthcare administrative data − EQ-5D-3 L

### Sample size

An anonymized cohort consisting of patients who received chemotherapy following curative surgery for early stage breast cancer (stage I-III) between January 1, 2007 and December 31, 2009 in Ontario, Canada was identified using the Ontario Cancer Registry (OCR) at the Institute for Clinical Evaluative Sciences (ICES), and used for the sample size calculation. Details of this methodology have been published previously [2]. Associations between the number of ED + H visits and centre, age, number of women treated, duration of chemotherapy and type of chemotherapy, were evaluated. We estimated that 20 of these institutions provide adjuvant therapy to at least 50 breast cancer patients annually and would agree to participate.

Two different simulation approaches were used to determine the sample size for the cRCT: (1) assuming an over-disperse Poisson model (negative binomial distribution) and using summary data from the 2007–2009 ICES cohort, we selected the top 25 cancer treatment centres by patient volume (top-25 cohort) to maintain estimate stability; and (2) using the actual data from the cohort and randomly allocating the 20 largest centres equally to control or treatment arm within 5 strata based on the number of women treated at each centre. With the first model-based approach, we applied a design effect to the number calculated assuming independence. The intra-class correlation for the top-25 cohort was estimated at 0.028. In the second actual data approach, we reduced the observed visits by a fixed pre-specified percentage ranging from 20 to 40%.

Both approaches resulted in similar estimates. With approximately 73 women per centre (total sample size, 1460) from 20 centres, we would achieve 80% power to detect a 33% reduction in the number of ED + H visits, with a one-sided alpha = 2.5%. For the secondary patient-reported outcomes sub-study (PRO sub-study), at least 25 patients per centre (total sample size: 500) need to be enrolled for 80% power (one-sided alpha = 2.5%) to detect a treatment effect size of 0.35 standard deviations.

### Cluster randomization

A covariate constraint-based randomization methodology proposed by Moulton [21] was used to randomly allocate the 20 participating centres to either intervention arm or control arm using a healthcare administrative dataset for early stage breast cancer patients diagnosed between January 1, 2010 and June 30, 2013 who received either adjuvant or neoadjuvant chemotherapy. The approach of Sismanidis et al. [22] was used to define the measure of imbalance in choosing the optimal allocation scheme. In brief, centres were grouped by the average ED + H visits per patient resulting in 14,000 unique unrestricted arrangements to force balance between the strata. Additional covariate-adjusted restrictions were applied to the groupings to achieve the acceptable mean difference between groups. One allocation scheme is then selected at random among all possible randomizations that achieve the best possible balance among the intervention arm and control arms followed by random assignment of study arms. Covariates considered in the model included patient volume, acute care visits, Charlson comorbidities, rurality, stage, regimen, facility type, nursing model, proportion of non-English speaking patients.

### Study participants

All women 18 years of age or older with a diagnosis of early stage (I-III) breast cancer starting treatment with adjuvant or neoadjuvant chemotherapy at one of the 20 participating cancer centres during the intervention period are considered eligible to participate in the study. The rate of ED + H visits has been shown to be higher in the routine care population than in randomized clinical trials of adjuvant chemotherapy, where rates of ED + H are rarely or never reported [5, 6, 23–25]. As such, patients currently receiving an investigational agent as part of a clinical trial are excluded from participation. However, there are few interventional trials expected to be accruing in the adjuvant breast cancer setting in Ontario during our trial. In addition, patients participating in the PRO sub-study must have the ability to understand and provide written informed consent, have language and literacy skills consistent with completing study questionnaires, and willingness to participate in the study and complete PRO questionnaires.

### Intervention nurses

As routine follow-up practices, nursing models and institutional structures vary across centres, participating centres are able to adapt the delivery of the intervention to their caseloads and individual contexts, consistent with the pragmatic design. However, core components of the intervention (booklets, call scripts, questionnaires, and call schedules) will be standardized across institutions. Intervention calls are delivered by local designated registered nurses with experience in symptom management who have undergone protocol-specific training on the toxicity management toolkit, including: Symptoms Self-Management Booklet - Healthcare Provider Edition (Additional file 1) and Telephone Follow-up Script (Additional file 2), through an Intervention Delivery Training Webinar prior to delivering any study calls.

### Intervention arm

**Fig. 2:**
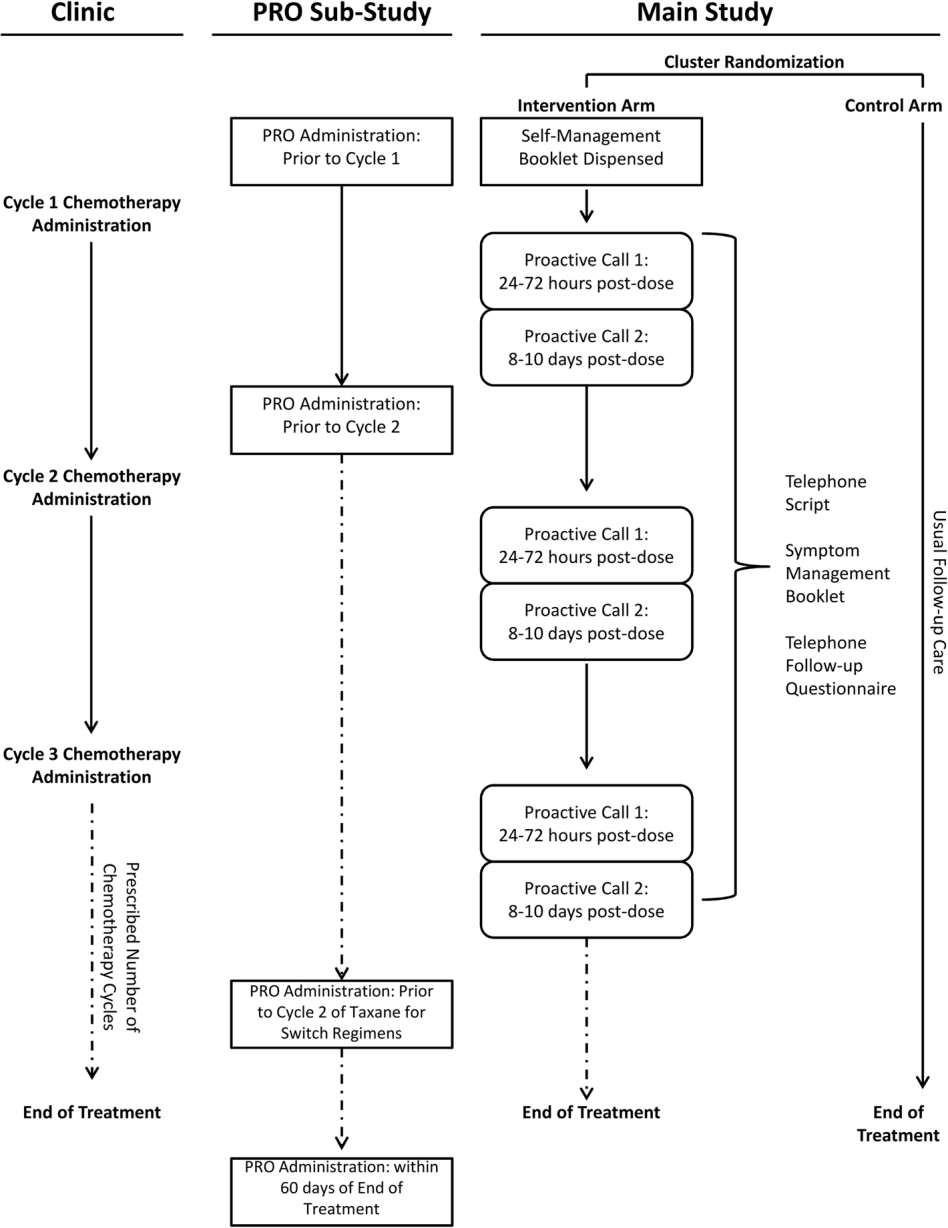
Overview of procedures for main study and patient reported outcomes (PRO) sub-study

Centres randomized to the intervention arm will offer the proactive telephone symptom management program, based on the findings of the AToM Pilot [13], to all patients beginning adjuvant or neoadjuvant chemotherapy for early stage breast cancer during the intervention period, and for the duration of their chemotherapy; patients receiving treatment exclusively with hormonal agents and/or targeted therapies are excluded. Participants in the intervention arm receive a copy of the Symptoms Self-Management Booklet- Patient Edition (Additional file 3) prior to initiating chemotherapy. Each participant receives two structured follow-up calls by the intervention nurse at their cancer centre during each chemotherapy cycle on the following schedule: (1) between 24 to 72 h after start of that cycle, and (2) between 8 to 10 days after start of each cycle (Fig. 2). For patients receiving treatment with weekly paclitaxel, calls are scheduled using a 3-week period as a cycle. Centres are also asked to track the number of patients who do not wish to receive the follow-up calls, as well as patients who wish to opt-out of receiving further calls or are lost to follow-up. To ensure a systematic symptom assessment occurs, the intervention nurses utilize the Telephone Follow-up Questionnaire (Additional file 4) during the calls, which addresses nine common chemotherapy-related toxicities: (1) nausea, (2) vomiting, (3) mouth and throat sores, (4) pain, (5) aching joints and aching muscles, (6) loose and watery stools, (7) shivering or shaking chills, (8) constipation, and (9) fatigue or tiredness. The intervention nurse provides standardized symptom management guidance using the Symptoms Self-Management Booklet - Healthcare Provider Edition (Additional file 1) and the Telephone Follow-up Script (Additional file 2). Additional unscheduled calls can occur 24 h following a scheduled call to follow-up on symptoms or to provide additional support at the discretion of the care team.

### Control arm

Centres randomized to the control arm continue to provide routine symptom management as is standard of care at their institution; routine care varies by institution. Centres allocated to the control arm do not have access to the intervention materials for the duration of the study (patient and provider editions of the Symptom Self-Management Booklets, Telephone Script, Telephone Follow-up Questionnaire and Intervention Delivery Training Webinar).

### Duration of intervention

To ensure the target accrual is achieved (approximately 73 patients per cluster), the duration over which the intervention is offered depends on the historic incident case load of participating institutions. For centres that historically see less than 150 new breast cancer cases who receive adjuvant or neo-adjuvant chemotherapy annually, all patients initiating chemotherapy in a 12-month accrual period will be included in the analysis. Likewise, for participating centres that historically see 150 or more patients starting adjuvant or neo-adjuvant chemotherapy annually, all patients beginning adjuvant or neo-adjuvant chemotherapy within a 6-month period will be included in the analysis. Patients who begin treatment during this time continue to receive the intervention until their course of chemotherapy is complete.

### Primary outcome

At the cluster-level, the summary measure is the mean number of ED + H visits per patient during the at-risk time defined as the time on chemotherapy treatment plus 30 days following the last chemotherapy delivery. The patient-level primary outcome is the number of ED + H visits during the at-risk time. Healthcare administrative data housed at ICES in Toronto, Ontario will be used to determine this value for each patient. All patients with early stage breast cancer at the participating centres who initiated adjuvant or neo-adjuvant chemotherapy during the intervention period will be identified from the New Drug Funding Database (NDFP) and/or Activity Level Reporting (ALR) database which include information on drugs received, dates of treatment and institution where treatment was given. The Ontario Cancer Registry (OCR) and the Collaborative Stage Database (CSD) will be used to confirm the patient has early stage breast cancer. The National Ambulatory Care Reporting System (NACRS) and Canadian Institutes for Health Information Discharge Abstract Database (CIHI-DAD) will be used to obtain information on ED visits and hospitalizations, respectively. Details of this methodology have been described previously [2].

### Patient reported outcomes sub-study

A sub-set of approximately 25 consecutive, consenting patients enrolled at each participating centre take part in a PRO sub-study to assess the effect of the telephone intervention versus control on secondary outcomes. Severity of treatment side effects is measured using the National Cancer Institute Patient Reported Outcomes version of the Common Terminology Criteria for Adverse Events [26, 27] self-report tool, a symptom severity index, and by the Edmonton Symptom Assessment System [28] already collected routinely in patients in regional cancer programs throughout Ontario. Self-efficacy or confidence in managing symptoms is measured using the Stanford Self-Management Self-Efficacy Scale, and general quality of life by the EQ-5D-3 L. [29] The Patient Health Questionnaire [30] and Generalized Anxiety Disorder [31] scales are used to evaluate major depression and anxiety, respectively. The Functional Assessment of Cancer Therapy-Breast scale [32] is used to evaluate physical, social and family wellbeing for breast cancer patients. Coordination and continuity of care is measured using the adapted Picker survey [33, 34]. Lastly, self-care during chemotherapy is assessed using the Leuven questionnaire (L-PaSC) [35].

Participants are required to complete the PRO questionnaires prior to the start of the first (baseline) and second cycles of chemotherapy, and within 60 days of the end of treatment. For patients receiving a chemotherapy regimen where they switch to a different drug part way through (usually addition of a taxane), there is an additional PRO collection prior to the start of the second cycle of the second drug except for the L-PaSC questionnaire which is only completed prior to second cycle of chemotherapy and within 60 days of the end of treatment. Questionnaires completed by patients participating in the PRO sub-study will be linked to administrative data housed at ICES using the Ontario Health Insurance Plan (OHIP) numbers provided by individually consenting patients.

### Economic analysis

All publicly available health system resources utilized by women receiving adjuvant or neo-adjuvant chemotherapy from initiation to the end of chemotherapy plus 30 days will be analyzed. Administrative databases will be used to identify and quantify these health system resources and will include ED (NACRS), hospitalization (CIHI-DAD), pre-hospital care (NACRS), and medications [NDFP, Activity Level Reporting (ALR)-systemic therapy database and the Ontario Drug Benefit Formulary (ODBF)]. Unit costs (2012 CAN$) for each of the resources identified, based on provincial (ED + H) and publicly available sources (medications), will be applied to the resources in order determine an overall cost per patient; a total cost per population and patient will be generated. We will conduct an incremental cost effectiveness analysis, comparing the incremental costs and the incremental outcomes between the intervention and control groups from the health system perspective. In this incremental analysis, effectiveness will be defined as hospitalization or ED visit avoided to generate the incremental cost per outcome avoided factoring in costs of the intervention. Sensitivity analyses will be conducted to determine the robustness of the results as well as cost and outcome drivers.

### Implementation Fidelity and adherence

Implementation fidelity will be assessed based on the core elements specified by Carrol et al. [36] To assess adherence to the intervention, the number of interactions with the patient by phone will be logged using a tracker provided in the toxicity management toolkit. Adherence is defined as 80% of toxicity management telephone calls completed (patient reached and counseling provided) within the protocol-specified call window. The adherence rate will be calculated per cycle and the mean calculated per patient and overall mean for all participants in the experimental arm. Participant responsiveness will be assessed by summing the number of phone calls attempted versus completed.

### Statistical analysis

A comparison between the control and intervention arms (10 clusters in each group; approximately 730 patients per arm) will be undertaken using a randomization test for the risk and rate differences which will take into account the restricted randomization. In addition, 95% confidence intervals for the differences will be calculated based on the randomization distribution. As a supportive analysis, the intervention and control groups also will be compared using Negative Binomial mixed effects regression modeling where we will consider the impact of patient-level baseline covariates, such as age, stage, comorbidities, socioeconomic status and treatment regimen, on the intervention effect. The at-risk period will be from the first day of chemotherapy until 30 days after the last treatment. Patient reported outcomes will be assessed using general linear mixed models to adjust for clustering of the individuals within centres and the between-centre variation and assess the impact of the intervention alone, and after adjusting for important patient-level baseline covariates. Multiple imputation within centres will be used to account for missed assessments.

### Ethical considerations

For centres randomized to the intervention arm, the intervention will be introduced in the centre as a process change as per quality improvement principles hence individual written informed consent will be waived [37]. Therefore, all eligible patients following the start of the intervention are offered telephone-based toxicity follow-up. This approach is supported by the fact that the intervention is associated with negligible risk of harm, substantially improves current standard of care where little to no proactive follow-up is performed after chemotherapy, and that individual consent is not practicable. For control centres employing their local standard of care, as above, informed consent is also waived. There is no additional respondent burden since there are no study-specific questionnaires to be completed by the overall study population.

Study-specific questionnaires will only be completed by those patients participating in the PRO sub-study, for which individual written informed consent is obtained. Sub-study enrollment is coordinated centrally by the Ontario Clinical Oncology Group (OCOG) Coordinating and Methods Centre located at the Juravinski Hospital in Hamilton, Ontario. Web-based secure patient registration is performed at the participating centres for all eligible, consenting patients. Participants are asked for their permission to collect and use their OHIP number for the purpose of linking data collected in the PRO sub-study to administrative databases. While provision of a patient’s OHIP number is voluntary, at least 500 consenting patients are needed to provide adequate statistical power for the analysis of secondary outcomes.

## Discussion

In this study, we will evaluate proactive telephone-based symptom management in patients receiving chemotherapy for early stage breast cancer using a pragmatic covariate constraint-based cluster randomized controlled trial design. Patients with early stage breast cancer tend to be treated with some combination of surgery and systemic therapy often involving chemotherapy. Recent studies suggest that ED visits and/or hospitalizations are very common among breast cancer patients receiving chemotherapy [1–6]; however, few cancer centres offer proactive approaches to the management of chemotherapy-related symptoms and availability of care outside regular clinic hours is limited. Thus far, most studies of chemotherapy-related toxicity interventions have utilized some form of telephone follow-up often coupled with a PRO tool to triage or facilitate the assessment or digital remote monitoring linked to clinician alerts. These studies show that a proactive approach to symptom management is feasible, acceptable to providers and can result in better symptom control but large scale studies evaluating population level impact on health service use are lacking [7–13]. Preliminary results from pilot testing of the AToM intervention at two Ontario cancer centres demonstrated feasibility and high patient and provider satisfaction with the intervention, warranting further evaluation impact of this approach on acute care utilization and patient reported outcomes in a large scale, pragmatic trial [13].

Use of a pragmatic trial design enables us to build on findings from our pilot work to examine how the intervention performs in a “real-world” setting and gain insights into potential barriers and facilitators for wide spread implementation of the intervention. As the organization of care to support symptom management during chemotherapy varies by institution, the design allows for the core elements of the intervention to be relevant in a variety of clinical scenarios while the delivery of the intervention is adaptable to different nursing models and institutional structures [38]. While the participating cancer centres are required to have evidence of sufficient caseload from historical administrative data to ensure that the required cluster-level sample size is achieved, participating centres range from small community hospitals to large, academic institutions. This helps to ensure that findings pertaining to barriers and facilitators for widespread implementation are applicable and translatable to the larger cancer community. One limitation is the focus on breast cancer only which will limit our ability to comment on experience in other disease sites. However, several of the previous smaller scale studies targeted a mixed group of patients and did not report differences by disease [39]. Digital tools with adaptive capabilities may be able to address the need for symptom monitoring customized to disease or regimen in the future.

To our knowledge, this is the first study to leverage routinely collected healthcare administrative data to analyze the primary outcome of a prospective, complex intervention trial in the cancer patient population. This helps to overcome resource barriers to participation by small community cancer centres associated with primary data collection and decreases the overall burden of primary data collection and the cost of performing the trial. While use of administrative data to identify and characterize ED + H visits within this population has been well documented [1, 2, 4], it is not without its challenges. It is difficult to ascertain who did or did not receive the intervention during the intervention period without collecting direct patient identifiers as it is not a standard variable. As such, all eligible patients at centres allocated to the intervention arm are expected to be offered the intervention during the intervention period. However, centres are required to track the number of patients opting out of receiving the intervention and reasons for opt-out to aid in contextual analysis of our findings. Administrative datasets provide a rich resource to examine healthcare utilization; however, the influence of issues with access to health care or individual patient care preferences in ED + H during chemotherapy are not well understood. By linking the quality of life and patient reported symptom severity outcomes for individually consenting patients in the PRO sub-study to the administrative datasets we hope to gain a greater understanding of the drivers of ED + H use.

## Additional files

Additional file 1: Symptom management guide - provider version. (DOCX 65 kb)

Additional file 2: Telephone script for follow-up calls. (DOCX 22 kb)

Additional file 3: Symptom management guide - patient version. (DOCX 62 kb)

Additional file 4: AToM telephone follow-up symptom tracking form. (DOCX 30 kb)

## Data Availability

Not applicable.

## Abbreviations

ALR: Activity Level Reporting
AToM: Ambulatory Toxicity Management
CDS: Collaborative Staging Database
CIHI DAD: Canadian Institutes for Health Information Discharge Abstract Database
cRCT: Cluster Randomized Controlled Trial
ED + H: Emergency Department Visits and Hospitalizations
ICES: Institute for Clinical Evaluative Sciences
L-PaSC: Leuven Questionnaire for Patient Self-Care
MOHLTC: Ministry of Health and Long-Term Care
NACRS: National Ambulatory Care Reporting System
NDFP: New Drug Funding Database
OCOG: Ontario Clinical Oncology Group
OCR: Ontario Cancer Registry
ODBF: Ontario Drug Benefit Formulary
OHIP: Ontario Health Insurance Plan
OICR: Ontario Institute for Cancer Research
PRO: Patient Reported Outcomes
