# Supplementary material for: Ambulatory Toxicity Management (AToM) in patients receiving adjuvant or neo-adjuvant chemotherapy for early stage breast cancer - a pragmatic cluster randomized trial protocol
Source: BMC Cancer. 2019 Sep 5;19:884. doi: 10.1186/s12885-019-6099-x (PMC6729066; doi:10.1186/s12885-019-6099-x)
Supplement: Supplementary file 1 — Symptom management guide - provider version. (DOCX 65 kb) [file 12885_2019_6099_MOESM1_ESM.docx]

Additional File 1

Symptom Management Guide for Early Stage Breast Cancer Patients receiving Adjuvant and Neoadjuvant Chemotherapy

Provider Version

Under Evaluation

This Symptom Management Guide has been developed to be used by health care providers to aid them in assisting their patients to prevent and manage their symptoms which are side effects of chemotherapy. All guides have been developed based on published guidelines for symptom management and telephone triage protocols.

The Symptom Management Guide includes management guides for the following symptoms:

| **Symptom** | **Page** |
| --- | --- |
| Nausea and Vomiting | 3-4 |
| Diarrhea | 5-6 |
| Constipation | 7-8 |
| Mouth and Throat Sores | 9 |
| Pain | 10-11 |
| Joint and Muscle Aches and Pain | 12 |
| Fatigue/Tiredness | 13 |

**Remember to reassure patients that the majority of the symptoms they experience as a result of chemotherapy are temporary and will improve with time.**

**Nausea and Vomiting Assessment & Management**

Assessment

- Level of severity for vomiting on the NCI PRO-CTCAE tool
- Characteristics of vomitus (i.e.colour, assess for blood, fecal contents, and amount), and how many days
- Is the patient taking their prescribed anti-emetics correctly (adherence)?
- Any symptoms of severe dehydration (i.e. decreased urinary output, dizziness, weakness, fainting, and dry mouth/thirst)
- Adequate oral intake (food and specifically fluid)?
- Assess whether related to chemotherapy
- Ask about heartburn or severe epigastric pain
- Any diarrhea or abdominal pain, or distention (rule out bowel obstruction and other causes for vomiting)

Care Map

| **Mild Nausea and Vomiting** | **Moderate Nausea and Vomiting** | **Severe Nausea and Vomiting** |
| --- | --- | --- |
| **Mild Severity NCI PRO-CTCAE**  **1-2 episodes of vomiting in 24 hours** | **Moderate Severity NCI PRO-CTCAE**  **2-5 episodes of vomiting in 24 hours**  **Dry mouth or excessive thirst**  **Able to take fluids- intake decreased** | **Severe Score NCI PRO-CTCAE**  **>6 episodes of vomiting in 24 hours**  **Signs of dehydration**  **No significant fluid intake**  **Gross bleeding in vomitus**  **Signs of bowel obstruction** |
| ***Non-pharmacologic*** | | |
| - Limit spicy, fatty or excessively salty or sweet foods - Avoid strong odours - Use small, frequent, bland meals and snacks - Sip water and other fluids - Consume food/liquids cold or at room temperatures to decrease odours - Sit upright or recline with head elevated for 30-60 minutes after meals | **Same as mild PLUS:**   - Consider making patient NPO until emesis resolves - Oral fluid and electrolyte replacement with over the counter rehydration solution - Advise patient to call if vomiting continues | **Need for urgent assessment and parenteral hydration.**  **Need for emergency action if abdominal distenstion, abdominal pain, severe epigastric pain, any blood, or fecal material in vomitus.** |
| ***Pharmacologic*** | | |
| - Discontinue any unnecessary medications that may be contributing to nausea and vomiting - Consider adding anti-nausea medications such as (dosing below): - Prochlorperazine, - Domperidone - Metoclopramide - Olanzapine - Nabilone - Dimenhydrinate - Ondansetron - Palonosetron - Aprepitant | **Same as mild PLUS:**   - Consider changing other medications to non-oral eg. intravenous, sc - Titrate anti-emetics to maximum tolerated dose - Prescribe anti-emetics on a regular dosing schedule with breakthrough dose - For anticipatory nausea or vomiting, give lorazepam prior to treatment - Prescribe olanzapine 2.5 mg po once or twice daily - For delayed nausea, consider extending duration of dexamethasone following chemotherapy beyond 24 hours - For heartburn symptoms, consider famotidine PO or a PPi - Review anti-emetics prior to next chemotherapy |  |

Medication Dosing:

- Prochlorperazine 10mg po/iv q8h
- Domperidone 10mg po TID to QID
- Metoclopramide 10 mg po QID prn
- Olanzapine 2.5-5 mg po QID prn
- Nabilone 1-2 mg po BID prn
- Dimenhydrinate 25-50mg po/sc/iv q4h
- Ondansetron 8mg po/sc/iv q8-24h, granisetron 1mg po BID or 1mg iv OD immediately following chemotherapy
- Palonosetron 0.5 mg po pre-chemo/0.25 mg IV pre-chemo
- Aprepitant 125/80/80 mg po Tri-Pack pre-chemo
- Lorazepam 1-2mg po/sl/iv/sc

**Diarrhea Assessment & Management**

Assessment

- Level of severity on NCI PRO-CTCAE tool
- Stool-colour, consistency, last usual bowel movement and any bleeding
- Onset, number of stools (frequency in last 24 hours) and how many days
- Adequate fluid intake to replace fluid loss
- Any fever, abdominal pain or cramping
- Any symptoms of dehydration (decreased urine output, thirst/dry mouth, dizziness, feeling faint, weakness, or racing heart)
- Use of medications and herbals
- Consider etiology including checking for *C. Difficile,* if appropriate (i.e. recently hospitalized)

Care Map

| **Mild Diarrhea** | **Moderate Diarrhea** | **Severe Diarrhea** |
| --- | --- | --- |
| **Mild Severity NCI PRO-CTCAE**  **Increase to 2-3 loose stools a day over pre-treatment movements** | **Moderate Severity NCI PRO-CTCAE**  **4-6 loose stools in past 24 hours and moderate cramping** | **Severe Score NCI PRO-CTCAE**  **>7 stools in past 24 hours**  **Signs of dehydration**  **Any fever or gross bleeding**  **Abdominal pain/severe cramping** |
| ***Non-pharmacologic*** | | |
| **Dietary modifications**:   - Eat small frequent meals - Limit caffeine, fried, greasy foods and dairy products - Avoid sorbitol containing foods - Limit/avoid foods high in ***insoluble*** fibre (wheat bran, fruit and vegetable skins, nuts and seeds, dark leafy greens) - Include foods high in ***soluble*** fibre (barley, potatoes, bananas, applesauce) - Avoid hyperosmotic liquids - Increase fluid intake | **Same as mild PLUS:**  Consider parenteral hydration  **Fluid intake :**   - Consider oral rehydration solution (homemade or commercially available) | - Parenteral hydration recommended - Arrange urgent assessment and review |
| ***Pharmacological*** | | |
| - If perianal skin is inflamed or excoriated consider topic corticosteroid cream for 1-2 days - Loperamide | - Try loperamide, if not successful at controlling diarrhea consider switching to diphenoxylate/ atropine (2.5/0.025 mg tablets) | - If loperamide or diphenoxylate/atropine not successful at controlling diarrhea consider:   - Opioids if patient not already on them   - Octreotide sc |

- Loperamide (2 mg tabs or 2mg/15 ml solution)
  - 2mg orally after each loose stool, up to 16 mg/day
  - For chronic diarrhea (or for diarrhea related to Irinotecan), regular BID dose can be used based on effective 24 hour dose plus 2mg after each loose BM (32 mg/day max)
- Diphenoxylate/atropine (2.5/0.025 mg tabs) – 1-2 tabs orally prn up to 4 times/day (max 20 mg/day of diphenoxylate)
- Octreotide 50-600 mcg per day sc BID – TID

Homemade Rehydration Solution:

Mix:

- 1 teaspoon salt
- 3 tablespoons of sugar
- 4 cups of water

**Constipation Assessment & Management**

Assessment:

- Usual routine and when was last bowel movement (more than 3 days since last BM)
- Description of stool (i.e. color, hardness, odor, straining, or blood)
- Any abdominal pain or vomiting
- Taking medication that causes constipation (i.e. analgesics, anti-depressants, some nausea meds such as ondansetron or granisetron)
- Use of medication for constipation
- Adequate food and fluid intake
- Rule out bowel obstruction (abdominal pain, abdominal bloating, fecal material in vomit, or persistent vomiting)

Care Map

| **Mild Constipation** | **Moderate Constipation** | **Severe Constipation** |
| --- | --- | --- |
| **Mild Severity NCI PRO-CTCAE**  **BM no less than 2 days ago** | **Moderate Severity NCI PRO-CTCAE**  **Less than 3 days since last BM** | **Severe Score on NCI PRO-CTCAE**  **Signs of Bowel Obstruction**  **No BM for more than 3 days**  **Acute distress** |
| ***Non-Pharmacologic*** | | |
| - Encourage fluid intake - Encourage ambulation as tolerated - Attempt toileting 30-60 minutes following ingestion of a meal to take advantage of the gastro-colic reflex | **Same as mild**  **Ask patient to call back if not resolved in 24 hours** | **Arrange for urgent assessment** |
| ***Pharmacologic*** | | |
| - Sennosides – Stool Softener - Bisacodyl - PEG 3350 (e.g., Restoralax®, Lax-A-Day®, etc.) - Lactulose - Milk of Magnesia | **Same as mild PLUS**   - Suppositories - Enemas | **Same as mild and moderate PLUS**   - Fecal disimpaction - Picosulfate dodium-magnesium oxide-citirc acid - Methylnaltrexone (if patient is taking regular opioids) |

Sennoside – Up to 4 tablets po BID prn

Bisacodyl – 1 per rectum OD prn

Lactulose –15-30 cc po TID prn

Milk of Magnesia – 15-30 cc po TID prn

PEG 3350 – 1 dose po OD prn

Initial 3 day trial of methylnaltrexone (only for patients taking regular opiods)

- If no bowel movement for 48 hours, give methylnaltrexone subcutaneously 8mg if 38-62kg or 12mg if 62-114kg
- Methylnaltrexone is considered effective if a bowel movement occurs within 4 hours after injection.
- The same dose can be repeated every 24 hours for 2 days if necessary. But after this point, if there is no bowel movement, methylnaltrexone is unlikely to work for this patient at this time and no further doses should be given.

**Mucositis Assessment & Management**

Assessment

- Onset and how many days
- Current oral care regime including use of oral rinses
- Is there evidence of bleeding, blisters, or severe erythema
- Any evidence of thrush (white patches on mucous membranes)
- Any fever (>38.3 degrees Celsius 101 degrees Farenheit)
- Are they able to drink or eat and is fluid intake adequate

Care Map

| **Mild Mucositis** | **Moderate Mucositis** | **Severe Mucositis** |
| --- | --- | --- |
| **Mild severity NCI PRO-CTCAE**  **Painless ulcers**  **Able to eat and drink** | **Moderate Severity NCI PRO-CTCAE**  **Painful erythema, edema**  **Oral ulcers** | **Severe Score NCI PRO-CTCAE**  **Any fever**  **Blisters coalescing/severe pain**  **Difficulty swallowing, eating or drinking**  **Signs of dehydration** |
| ***Non-pharmacologic*** | | |
| **General oral care**:  Bland rinse solution prepared once daily and not refrigerated  **Dietary modifications:**   - Soft, smooth foods - Avoid foods with abrasive, rough, tart, salty, spicy, acidic, very hot or cold foods | **Same as mild**   - Consider extra soft/pureed diet - If only liquids are tolerated, choose high calorie, protein fluids every 2 hours - Oral commercial nutrition supplements are recommended - Dietician consult | - **Arrange urgent assessment and review** |
| ***Pharmacological*** | | |
| - Topical anesthetic* | - Consider oral non-opioid or opioid analgesics if topical anesthetics are not effective for pain relief | - Patient controlled analgesia with opiods - Consider parenteral analgesics |

Bland Rinse Solution:

Mix:

- 1 teaspoon salt
- 1 teaspoon baking soda
- 1 cup water

Topical anaesthetics: Example- Orajel, 2% viscous Lidocaine

Thrush – Nystatin 100,000 U/mL, Fluconazole tablets po

Oral analgesics recommended starting doses

**Pain Assessment & Management**

Assessment

- Location, is it new, alleviating and aggravating factors, other symptoms accompanying pain, and number of days
- Assess patient’s current pain management regimen, including pain medications from other prescribers and over the counter medications
- Assess whether patient is compliant with pain medication and taking as prescribed
- Is pain sudden or acute onset or changed (i.e. new headache pain accompanied by visual disturbance)?
- Rule out spinal cord obstruction, cardiac causes of pain if new acute pain, or thrombosis

Care Map

| **Mild Pain** | **Moderate Pain** | **Severe Pain** |
| --- | --- | --- |
| **Mild Score on NCI PRO-CTCAE**  **Not interfering with function** | **Moderate Score on NCI PRO-CTCAE**  **Some interference in activities of daily living (ADL)** | **Severe Score on NCI PRO-CTCAE**  **Severe interference in ADL**  **Acute onset of new pain**  **Signs of untoward pain**  **Acute distress** |
| ***Non-pharmacologic*** | | |
| - Patient education about routine and breakthrough analgesia | **Same as mild** | **Arrange urgent assessment and review.** |
| ***Pharmacologic*** | | |
| - Acetaminophen - NSAIDS and COX2 inhibitors + gastric mucosa protection (eg. Proton pump inhibitors) - Weak opioids eg. ½ tablet oxycocet q4h prn | **Same as mild PLUS**  If a patient is opioid naïve:  (all doses are ORAL/PO)   - Morphine starting dose is usually 5mg q4h with 2.5-5mg q1h prn for breakthrough pain - For elderly or debilitated patients consider a lower starting dose of 2.5 mg q4h - Hydromorphone starting dose is usually 1mg q4h with 0.5-1mg q1h prn for breakthrough pain. For elderly or debilitated patients consider a lower starting dose of 0.5mg q4h - Oxycodone starting dose is usually 2.5mg (half tablet of a 5mg) q4h with 2.5mg q2h prn for breakthrough. The lowest dose oxycodone tablets available either in combination with acetaminophen or alone contain 5mg of oxycodone, equivalent to 5-10mg of morphine   If a patient is NOT opioid naïve   - Increase the patient’s immediate release preparation regular and breakthrough doses by 25% - Increase the patient’s regular sustained release opioid dose by 25%. PLUS change breakthrough dose to 10% of the regular 24h dose either q1-2h prn po or q30 min prn SC | - Consider pain consultation once pain crisis is resolved |

**Joint and Muscle Aches and Neuropathy Assessment & Management**

Assessment

- Onset, how many days, and severity
- Assess patient’s current pain management regimen including pain medications from other prescribers and over the counter meds
- Assess whether patient is compliant to pain medication and taking as prescribed
- Interference in mobility or function or in activities in daily living

Care Map

| **Mild Pain** | **Moderate Pain** | **Severe Pain** |
| --- | --- | --- |
| **Mild severity on NCI PRO-CTCAE**  **Mild parasthesis** | **Moderate severity NCI PRO-CTCAE**  **Sensory loss, mild weakness, no loss of function** | **Severe Score on NCI PRO-CTCAE**  **Severe sensory loss or weakness**  **Impaired mobility or function** |
| ***Non-pharmacologic*** | | |
| - Light exercises, stretching - Heating pads - Ensure adequate hydration - Protect hands and feet from extremes of hot and cold | **Same as mild** | **Urgent Assessment and Review** |
| ***Pharmacologic*** | | |
| - Acetaminophen - NSAIDs (e.g., Ibuprofen, Naproxen) – if platelets adequate | - Acetaminophen + Opioid combination product - NSAIDs (Prescription strength) – if platelets adequate - Gabapentin or Pregabalin | - Pure opioid (i.e. morphine) - Dexamethasone 4mg po OD x 4 days |

**Fatigue Assessment & Management**

Assessment

- Onset and how many days
- When considering the multiple possible etiologies, keep in mind that fatigue could be related to the malignancy itself, treatment for malignancy (chemotherapy, radiation or surgery), other illnesses, paraneoplastic syndromes, or psychosocial causes
- Rule out anemia, hypothyroidism, fluid and electrolyte imbalance, or depression
- Rule out cardiac condition (i.e. heaviness in chest, pain in arm or jaw, new profound fatigue, dyspnea, or palpitations)
- Rule out acute or sudden onset with fever: sign of infection
- Impairment in daytime functioning, sleeping more than 20 minutes in day
- Assess for sleep disturbance (early morning wakening, difficult to sleep, interrupted sleep)

Care Map

| **Mild Fatigue** | **Moderate Fatigue** | **Severe Fatigue** |
| --- | --- | --- |
| **Mild Severity NCI PRO-CTCAE**  **Minimal impairment** | **Moderate Severity NCI PRO-CTCAE**  **Some interference in daytime functioning** | **Severe Score NCI PRO-CTCAE**  **Severe interference in activities of daily life (ADL)**  **Daytime impairment in ADL**  **Any cardiac symptoms** |
| ***Non-pharmacologic*** | | |
| - Treat underlying causes - Advise patients that they can perform activities as tolerated - Advise walking if no contraindications-gradual to tolerance - Nutritional counseling (Pro-FT-1) - Optimize sleep using sleep hygiene strategies (Pro-FT-2) - Psychosocial support (Pro-FT-3) - Advise patient in pacing and balancing of activities and rest | **Same as mild** | **Same as mild PLUS**   - Consider offering home care services, if appropriate (Pro-FT-4) - Any cardiac symptoms-arrange for urgent review - Arrange for review at clinic if no acute onset or other signs of cardiac or low hemoglobin (i.e. new shortness of breath) |

Treat anemia, other biochemical abnormalities, or potential treatable causes of fatigue.

**References:**

1. Cancer Care Ontario. Symptom Management Practice Guides- Algorithms. 5 May 2014. <https://www.cancercare.on.ca/cms/one.aspx?portalId=1377&pageId=58189> [accessed: 3 Dec 2015].
2. Thames Valley Cancer Network. Guidelines on prevention and management of Chemotherapy and radiotherapy induced diarrhea. 16 Sept 2011. <http://tvscn.nhs.uk/wp-content/uploads/2014/09/Cancer-Chemotherapy-Guidelines-on-Prevention-Management-of-Chemo-RT-Induced-Diarrhoea.pdf> [accessed: 3 Dec 2015].
3. Pan Birmingham Cancer Network. Guideline for 24 Hour Telephone Advice for Patients Receiving Anti- Cancer Treatment. Jun 2012. <https://www.uhb.nhs.uk/Downloads/pdf/CancerPbTelephoneAdviceService.pdf> [accessed: 3 Dec 2015].
4. St. James Hospital Hematology and Oncology. Telephone Triage Guidelines. 2012. <http://www.stjames.ie/Departments/DepartmentsA-Z/M/MedicalOncology/DepartmentinDepth/Telephone%20Triage%20Guidelines.pdf> [accessed: 3 Dec 2015].
