# Supplementary material for: Ambulatory Toxicity Management (AToM) in patients receiving adjuvant or neo-adjuvant chemotherapy for early stage breast cancer - a pragmatic cluster randomized trial protocol
Source: BMC Cancer. 2019 Sep 5;19:884. doi: 10.1186/s12885-019-6099-x (PMC6729066; doi:10.1186/s12885-019-6099-x)
Supplement: Supplementary file 2 — Telephone script for follow-up calls. (DOCX 22 kb) [file 12885_2019_6099_MOESM2_ESM.docx]

**ADDITIONAL FILE 2**

**Telephone Script- Follow-Up Calls**

**Study Name**: A Pragmatic Cluster-Randomized Trial of Ambulatory Toxicity Management (AToM) in Patients Receiving Adjuvant or Neo-adjuvant Chemotherapy for Early Stage Breast Cancer

The AToM phone script is to be used during follow up telephone call to patient participating in the intervention 24 to 72 hours and 8-10 days following each chemotherapy treatment. No calls will be placed to patients who have chosen to opt-out during their initial introduction of the initiative by a member of their team of care. During this initial introduction to the intervention, discuss and record the patient’s preferred phone number for follow-up contact and their preferences regarding use of voicemail.

Script:

Call number the patient has provided.

**If no one answers and it goes to voicemail** – leave a message ONLY if the patient has provided permission to leave a message.

*Hi (insert patient’s name), this is (insert caller’s name), the nurse/pharmacist/study coordinator at (insert centre name). I am calling to check in with you regarding any symptoms you may have. Can you please call me back at (insert phone number).*

If the patient has NOT provided permission to leave a message, disconnect the call and try calling the patient again later in the day.

**If a person other than the patient answers:**

*Hello, may I please speak with (insert patient’s name)?*

**If the person on the phone is NOT the patient and they say the patient is NOT available*:***

If patient has not provided permission to leave message with other people answering calls at this number:

*Do you know when would be a better time that I could call them? (Wait for response)*

*Thank you. I will try calling again later.*

If patient has provided permission to leave message with other people answering calls at this number:

*This is (insert caller’s name), the nurse/pharmacist/study coordinator at (insert centre name). I am calling to check in on how Mr./Ms. (patient’s name) is feeling. Can you please ask (insert patient’s name) to call me back as soon as possible at (insert phone number).*

**If the patient answers or you are connected to the patient by the person who answers the phone:**

*Hi (insert patient’s name,)*

*This is (insert research coordinator’s name), the nurse/pharmacist/study coordinator at (insert centre name). I am calling to follow-up on how you are feeling and any symptoms you may have experienced since your last chemotherapy appointment.*

*Is this a good time to talk with you?*

If NO:

*Is there a time that works better for you, that I can call you?*

If YES:

*Thank you. I am going to assess any symptoms you may be experiencing and their severity.*

Fill out study forms – Telephone follow-up form

Discuss how the patient can manage any symptoms they have by using the tips listed in the “Patient Symptom Management Guide”.

If the patient, identifies symptoms that are listed under the “When to seek medical treatment” section of the “Patient Symptom Management Guide”, recommend that the patient seek further medical treatment (call their doctor, go to urgent care or the emergency department or call 911). Ask the patient specifically as to who, how, when they will take this urgent action-ask them to recall back to you and why this is urgent. Reinforce if needed.

After finishing forms and discussing symptom management:

*Can you summarize for me the actions you will take to manage the symptoms we discussed (Prompt: for each symptom discussed if needed). Ask the patient, “how confident are you that you can implement the strategies for managing these symptoms as discussed”.*

*How confident are you that you can implement the strategies discussed to manage your symptoms?*

Not at all I I I I I I I I I I I Totally

Confident 0 1 2 3 4 5 6 7 8 9 10 Confident

*( if confidence is low (>7) ask about barriers and problem solve with the patient alternate ways to manage symptom). Record this score and actions taken in your process notes.*

Not at all I I I I I I I I I I I Totally

Confident 0 1 2 3 4 5 6 7 8 9 10 Confident

*Do you have any other questions about what we have talked about?*

*If you have any further questions about what we have discussed today, please call me or contact your doctor. I will see you at your next chemotherapy appointment. Thank you for your time.*
