# Supplementary material for: Ambulatory Toxicity Management (AToM) in patients receiving adjuvant or neo-adjuvant chemotherapy for early stage breast cancer - a pragmatic cluster randomized trial protocol
Source: BMC Cancer. 2019 Sep 5;19:884. doi: 10.1186/s12885-019-6099-x (PMC6729066; doi:10.1186/s12885-019-6099-x)
Supplement: Supplementary file 3 — Symptom management guide - patient version. (DOCX 62 kb) [file 12885_2019_6099_MOESM3_ESM.docx]

Additional File 3

Symptom Management Guide for Early Stage Breast Cancer Patients receiving Adjuvant and Neoadjuvant Chemotherapy

Patient Version

Under Evaluation

This Symptom Management Guide has been developed to be used by patients to assist them in preventing and managing their symptoms which are side effects of chemotherapy. This symptom management guide is based on information found on the Cancer Care Ontario webpage- cancercare.on.ca, Canadian Cancer Society webpage – [www.cancer.ca](http://www.cancer.ca), and the American Cancer Society webpage – [www.cancer.org](http://www.cancer.org).

The Symptom Management Guide includes management guides for the following symptoms:

| **Symptom** | **Page** |
| --- | --- |
| Nausea and Vomiting | 3 |
| Diarrhea | 4-5 |
| Constipation | 6 |
| Mouth and Throat Sores | 7 |
| Pain | 8 |
| Muscle and Joint Aches and Pains | 9 |
| Fever | 10 |
| Fatigue/Tiredness | 11 |

**It is important to remember the symptoms you experience as a result of your chemotherapy are temporary.**

If you require urgent care, you should do one or more of the following:

- Call your cancer health care provider;

Doctor/Nurse Name:__________________________________

Phone Number:______________________________________

- Call TeleHealth Ontario (1-866-797-0000)
- Go to the nearest urgent care clinic;
- Go to the emergency room;
- Call 911.

**Nausea and Vomiting Symptom Management**

**What is nausea and vomiting?**

Nausea is a feeling of being sick to your stomach. This can lead to vomiting. Vomiting is when you empty the contents of your stomach through your mouth.

**IMPORTANT:**

It is easier to stop nausea from happening than to treat it. For this reason, it is important to take anti-nausea medications that your doctor gives you and follow the instructions on how to take it.

**What medications will be given to me to help my nausea and vomiting?**

Your doctor may give you a prescription for a type of drug called an **anti-emetic** to prevent or reduce nausea. It is important to take this medicine as directed. If the anti-emetic does not work or causes any problems you should speak with your doctor.

**When to contact your doctor or go to the hospital?**

- You cannot stop vomiting.
- Vomiting goes on for more than 24 hours after your treatment (even if you are taking anti-emetics as directed).
- When there is blood in your vomit.
- If you feel weak, dizzy or confused.

**How can I stop nausea and vomiting from happening and what can I do if it does happen?**

- Eat small amounts of dry foods like crackers, toast, dry cereals or bread sticks. Try to do this as soon as you wake up and every few hours during the day.
- Sip water and other liquids like flat ginger ale, sports drinks, herbal teas, broth during the day. Cool liquids may be easier to drink than hot or cold liquids.
- Suck on ice chips.
- Eat food that is served cold may help to take away the strong smells.
- Drink liquids through a straw to avoid strong smells.
- Eat small meals more often during the day and eat slowly.
- If cooking smells bother you, ask someone to make food for you.
- Try not to eat foods that are sweet, greasy, fried, spicy or that have strong smells.
- Rinse your mouth with club soda before and after meals.
- Keep your mouth clean by brushing your teeth at least twice a day.
- Try not to lie down for at least 1 hour after meals unless head is raised on a pillow.
- Wear loose clothing. Clothing that is tight around the waist can make nausea feel worse.

**Diarrhea Symptom Management**

**What is diarrhea?**

Diarrhea is loose or watery or soft bowel movements that happen more than 3-4 times a day.

**IMPORTANT:**

Some cancer treatments may cause loose, frequent stools.

**What medications will I be given for my diarrhea?**

Speak with your doctor about managing your diarrhea. They may suggest an over the counter medication, such as loperamide, to reduce diarrhea (examples: Pepto-Bismol or Imodium) or a prescription medication.

**When should I talk to my doctor or go to the hospital?**

If your diarrhea:

- Continues for more than 24 hours
- Causes pain in your stomach
- Causes cramping
- Contains blood
- You have more than 7 bowel movements per day

You should keep record of the number, amount and appearance of bowel movements for your doctor.

**What can I do to stop diarrhea or treat it?**

- Eat small meals and snacks often during the day.
- Drink plenty of non-carbonated, caffeine-free fluids like water, juice, sports drinks and clear broth. Warm or room temperature liquids may be easier to drink. Drink an extra cup of liquids for every watery bowel movement. This will help prevent you from becoming dehydrated.
- Avoid drinking through a straw as this will produce more gas in your bowels and make you uncomfortable
- Switch to low-lactose milk or soy beverage if drinking milk makes your diarrhea worse.
- Choose salty foods to get sodium back in your body that may be lost from having diarrhea.
  - Examples: soup, sports drinks, crackers and pretzels
- Choose foods that are high in potassium to replace lost potassium.
  - Examples: bananas, tomatoes, fruit juices and nectars, and sports drinks
- Choose foods that are low in insoluble fibre, which gives bulk to stool.
  - Examples: white bread and pasta, refined cereals, ripe bananas, mashed potatoes or potatoes without skins, meat, poultry and fish
- Limit foods and drinks that contain caffeine.
  - Examples: coffee, tea, chocolate and cola
- Limit greasy, fried, spicy or sugary foods.
- Stay away from nuts, seeds, dried peas and beans.
- Stay away from foods that are natural laxatives.
  - Examples: prunes and prune juice, rhubarb and papaya
  - Avoid sugarless gum and candies made with sorbitol, which acts like a laxative.
- With diarrhea, your skin may be at risk of breaking down. A sitz bath is a warm soothing soak for your perineal or bottom area that contains water, baking soda (sodium bicarbonate) or salt. A sitz bath cleanses, reduces the chances of infection and increases blood circulation to help with healing.

**How can I prepare a sitz bath?**

A sitz bath can be done using your bathtub at home, a plastic sitz bath that fits over a toilet seat that you can buy from the drug store, or a large basin. You can have a sitz bath 4 times a day more or less, depending on your comfort. To prepare a sitz bath:

1. Wash your hands.
2. Fill plastic sitz bath, basin or your bathtub with enough warm water to soak your bottom.
3. Add 1 to 2 tablespoons of baking soda, or 1 to 2 teaspoons of salt to the water (enough to make the water feel silky).
4. Do NOT add bath oils or anything else to the water.
5. Soak your bottom in the sitz bath for 10-15 minutes.
6. GENTLY pat dry your bottom with a clean, soft towel or allow the area to air dry.
7. Clean the plastic sitz bath after each use.

**Constipation Symptom Management**

**What is constipation?**

Constipation is when you do not have regular bowl movements or you are passing of hard or difficult stool which causes discomfort or pain.

**IMPORTANT:**

Changes in your bowel habits can be a result of cancer and its treatment, certain medications (such as anti-nausea medications like ondansetron and granisetron), changes in your eating habits (including how much fluid you are taking in) or being less active.

**What medications may I be given to help with constipation?**

Your physician may recommend a stool softener or laxative (examples: Senekot, Dulcolax).

**When should I talk to my doctor or go to the hospital?**

- You have not had a bowel movement in 3 days.
- You have not had a bowel movement within 1 or 2 days of taking a laxative.
- You have cramps or vomiting that does not stop.
- You have blood in your stool.

**What can I do to stop and manage constipation?**

- Slowly add more fibre to your diet.
  - Examples of high fibre foods: whole grain breads and cereals, fruit, vegetables, legumes, dried fruit, seeds and nuts.
- Drink lots of fluids during the day to help move the fibre through you. If It is hard for you to drink a lot at one time, take small sips of liquids often or eat fruits. Try: water, fruit and vegetable juices, teas, broth, soup or lemonade.
- Choose a cereal that contains more than 4 grams of fibre per serving.
- Consume natural laxatives
  - Examples: rhubarb, papaya, 50ml (1/4 cup) prunes or 125ml (1/2 cup) prune juice.
- Add small amounts of bran to cooking or baking.
- If you can, be more physically active.

**Mouth and Throat Sores Symptom Management**

**What are mouth and throat sores?**

Mouth sores and throat sores are small cuts or ulcers. They can look red or white in the center.

**IMPORTANT:**

You may get mouth sores 1 or 2 weeks after chemotherapy treatment and they may take 2 to 4 weeks to heal or get better.

**What medications will be given to help stop or treat mouth sores?**

If your mouth or throat sores are making it hard to eat or drink, talk to your health care team. They may suggest an oral topical anesthetic (such as an Orajel) or an analgesic (medication that reduces or takes away pain).

**When should I talk to my doctor or go to the hospital?**

- You cannot take medications because of mouth sores.
- You cannot eat or drink because of mouth sores.
- You have an oral or tympanic (ear) temperature of 100.9 degrees Fahrenheit (38.3 degrees Celsius)

**How can I stop mouth sores or what can I do to make them better?**

- Rinse your mouth a few times a day.
  - Your health care team may suggest a rinse to you.
  - You can try mixing 5ml (1tsp) baking soda with 250ml (1 cup) of water.
- Sip on club soda.
- Eat soft and bland foods that are lukewarm or cool.
  - Some types of these foods include: creamed soups, mashed potatoes, yogurt, eggs, custards, puddings, cooked cereals, ice cream, casseroles, milkshakes and commercial nutrient supplements.
- Try not to eat acidic fruits and juices like orange or grapefruit juice.
- Try not to drink alcohol or eat foods that are tart, salty or spicy as they may bother your mouth and make sore more painful.
- Try not to drink or eat foods that are either very hot or very cold as they may make mouth or throat sores more painful.
- Use a straw for drinking to reduce contact between the drink and your mouth.

**Pain Symptom Management**

**Remember**: Do not lose hope. Cancer pain can be controlled or made better.

**What is pain?**

People talk about pain as hurting somewhere in the body. Pain is your body’s way of telling you that something is wrong.

**IMPORTANT:**

Cancer pain can be caused by the cancer itself, medical tests or procedures, and cancer treatments. Your health care team will find the cause of the pain and find steps to make it better. Physical pain and emotional pain can go hand-in-hand. It is important to talk to your doctor, your family and friends about both physical and emotional pain.

**What medications will I be given for my pain?**

Your doctor may prescribe you one of three types of drugs to help you with your pain. These drugs are called non-opioids, opioids and adjuvant drugs.

- Non-opioids – help mild to moderate pain. Non-opioids include acetaminophen (Tylenol), non-steroidal anti-inflammatories (NSAIDs) and corticosteroids (dexamethasone).
- Opioids – help moderate to severe pain. Opioids include codeine, oxycodone, methadone, hydromorphone, fentanyl and morphine.
- Adjuvant Drugs – help to control pain and symptoms. Adjuvant drugs include drugs for nerve pain, anti-anxiety drugs, anti-depressants and muscle relaxants.

It is very important to take pain medications as prescribed.

**When to should I talk to my doctor or go to the hospital?**

- You develop new or severe pain
- You have trouble waking up or staying awake
- You become confused

**What can I do to help my pain?**

- Take medication at the times prescribed by your doctor.
- If possible, keep up your usual exercise. Gentle activities may help you feel better and reduce your pain.
- Try to get enough sleep.
- Tell your health care provider about all of the medications and supplements you are taking to make sure they are safe to take along with the other medications you are taking and they are not affecting how well your pain medication works.
- Keep track of your pain through a pain diary. Keep track of the medication you take and the other ways you get help for your pain. You should track how bad is the pain, how long it lasts and how long it takes for medicine to help the pain get better.
- Talk to your doctor about symptoms related to your pain medication.

**Joint and Muscle Aches and Pains Symptom Management**

**What are muscle and joint aches and pains?**

Muscle pain can be described in two ways:

1. A deep, dull and steady ache
2. A quick, random and sharp ache

The pain may be focused all over the body or just in one specific area. Pain can range from mild and manageable to severe and debilitating. Severe muscle and joint pain are expected to improve over the next week.

Some chemotherapy treatments used in breast cancer patients may cause joint and muscle aches and pains. Neulasta and Neupogen (drugs used to prevent and treat infections in patients receiving chemotherapy) may also cause muscle and joint pain.

**Remember**: Do not lose hope. Most muscle and joint pain caused by breast cancer treatments goes away after you stop taking medications.

**What medications will be given to me to help my joint and muscle aches and pains?**

Your doctor may prescribe or recommend an anti-inflammatory, steroids or an opioid pain killer to help relieve muscle and joint aches and pains. Your doctor may consider altering your chemotherapy treatment to relieve aches and pains.

**When to contact your doctor or go to the hospital?**

- Distressing pain despite opioid medication

**How can I stop joint and muscle aches and pains from happening and what can I do if it does happen?**

- Use hot or cold compresses or a combination of the two to ease discomfort in a specific area
- Take warm baths to sooth pain all over
- Consider massage or acupuncture to relieve pain (speak with your doctor first).
- Eat a healthy diet that includes enough calcium and vitamin D to keep your bones strong.
- Maintain healthy weight to ease stress and strain on your joints
- Exercise regularly.
- Rest to allow your muscles to relax and recover.
- Take pain medication as prescribed by your doctor.
- Keep a pain diary. Keep track of the medication you take and the other ways you get help for your pain. You should track how bad the pain is, how long it lasts and how long it takes for medicine to help the pain get better.

**Fever Symptom Management**

**What is a fever?**

A fever is when you take your temperature (in your ear or mouth) and it is **100.9 degrees Fahrenheit (or 38.3 degrees Celsius) or higher**.

**IMPORTANT:**

Fevers can be a sign of infection (bacterial or viral). They may also be the result of inflammatory illness (like Crohn’s or colitis), drug reactions or tumour growth. People being treated with chemotherapy are more likely to get infections as chemotherapy lowers the number of white blood cells, which we need to fight infections.

**What medications will I be given to treat a fever?**

Your doctor may suggest acetaminophen (Tylenol) or other medications for fever. These should only be taken if your doctor tells you to do so.

**When should I talk to my doctor or go to the hospital?**

- You have a fever (100.9 degrees Fahrenheit or 38.3 degrees Celsius or higher)
- You have shaking chills
- You cannot take fluids
- You have 2 or more symptoms listed in the symptoms to watch for list.

Symptoms to watch for are:

- Skin rashes
- A new area of redness or swelling
- Pus or yellow discharge from an injury or other location
- New cough or shortness of breath
- New abdominal pain
- Burning or pain when urinating
- Sore throat
- Patient is confused (don’t know where they are, forgetful or not making sense)

**How should I treat a fever?**

- If you start feeling warm or cold, check your temperature every 2 to 3 hours. Keep a record of your temperature readings.
- Drink plenty of fluids.
- Examples: water, fruit juices, sports drinks, popsicles and soup.
- Get enough rest.
- Cover yourself in a blanket if you feel chilly.
- Cover yourself in a sheet if you feel hot.
- Place a cold wash cloth on your forehead if you feel hot.
- Contact healthcare provider for advice.

**Fatigue/Tiredness Symptom Management**

**What is cancer related fatigue?**

Fatigue is the feeling of being very tired and exhausted that does not go away with rest or sleep.

**IMPORTANT:**

Chemotherapy, hormonal drug therapy, biological therapy and targeted therapy can all cause fatigue.

Most people get fatigue during treatment for cancer. You may feel weak, heavy or slow and have trouble concentrating and remembering things. Other things that can add to your fatigue include low blood counts, the effort of going to so many medical appointments, as well as worry and stress. Dealing with side effects, such as pain, trouble sleeping ad changes to diet, can also make you feel very tired.

**What medications will I get for fatigue?**

Tell your health care team when you feel most tired, when you have energy and if sleeping helps you feel rested or not. It’s possible that you may need medicine, a nutritional supplement or a blood transfusion.

**When should I talk to my doctor or get medical help?**

- You are too tired to get out of bed for more than 24 hours.
- You become confused, dizzy, cannot think clearly, or fall.
- You feel out of breath and your heart is racing after a small activity.
- Your fatigue suddenly gets much worse.
- You have unexplained bleeding or bleeding that does not stop.
- You have anxiety, depression or feelings of not coping well.

**What can I do to help with my fatigue?**

- Keep track of your energy levels during and after treatment in a journal. Write down when you feel most tired and when you have more energy. Try to schedule appointments or activities you enjoy when you have the most energy.
- Plan ahead. Do the things that mean the most to you first. Plan rest periods before activities. Ask about a flexible work schedule or reduced hours, if you are still working during your treatment.
- Drink a lot. Drink plenty of fluids and eat when you have the most energy. It helps to have 5 or 6 small snacks throughout the day INSTEAD of 3 large meals.
- Be active. Gentle activity can give you more energy.
- Rest when you need to. Most people find it helpful to take short naps of 10 to 15 minutes rather than longer naps during the day. Save you longest sleeps for at night.
- Let others help. Ask friends and family to help you when you feel most tired.
- If you don’t have the energy to meet in-person, keep in touch by phone, e-mail or social media.
