# Supplementary material for: Ambulatory Toxicity Management (AToM) in patients receiving adjuvant or neo-adjuvant chemotherapy for early stage breast cancer - a pragmatic cluster randomized trial protocol
Source: BMC Cancer. 2019 Sep 5;19:884. doi: 10.1186/s12885-019-6099-x (PMC6729066; doi:10.1186/s12885-019-6099-x)
Supplement: Supplementary file 4 — AToM telephone follow-up symptom tracking form. (DOCX 30 kb) [file 12885_2019_6099_MOESM4_ESM.docx]

**Additional File 4**

**AToM TELEPHONE FOLLOW-UP FORM**

**
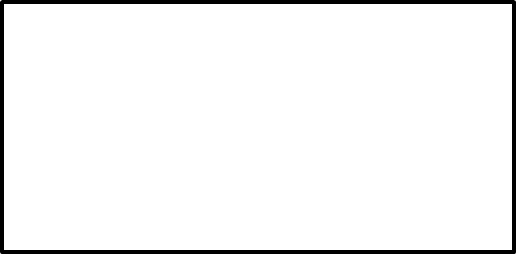
**Date of Assessment: ____________________________________

***Instructions:***

***Please ask the patent to evaluate the presence and severity of symptoms based on the last 7 days.***

***Please complete the symptom assessment and provide the symptom management recommendations where necessary.***

| **Chemotherapy Toxicity** | **Frequency** | **Severity** | **Were Recommendations Made?** | **Recommendations Made** |
| --- | --- | --- | --- | --- |
| **Nausea** | - None - Rarely - Occasionally - Frequently - Almost Constantly | - Mild - Moderate - Severe - Very Severe | - Yes - No | - Non-pharmacological - Pharmacological - Patient should speak to health care provider about symptoms at next clinic visit - Patient should seek immediate assistance (ER) |
| **Vomiting** | - None - Rarely - Occasionally - Frequently - Almost Constantly | - Mild - Moderate - Severe - Very Severe | - Yes - No | - Non-pharmacological - Pharmacological - Patient should speak to health care provider about symptoms at next clinic visit - Patient should seek immediate assistance (ER) |
| **Diarrhea** | - None - Rarely - Occasionally - Frequently - Almost Constantly | - Mild - Moderate - Severe - Very Severe | - Yes - No | - Non-pharmacological - Pharmacological - Patient should speak to health care provider about symptoms at next clinic visit - Patient should seek immediate assistance (ER) |
| **Chemotherapy Toxicity** | **Frequency** | **Severity** | **Were Recommendations Made?** | **Recommendations Made** |
| **Constipation** | - None - Rarely - Occasionally - Frequently - Almost Constantly | - Mild - Moderate - Severe - Very Severe | - Yes - No | - Non-pharmacological - Pharmacological - Patient should speak to health care provider about symptoms at next clinic visit - Patient should seek immediate assistance (ER) |
| **Mouth and Throat Sores** | - None - Rarely - Occasionally - Frequently - Almost Constantly | - Mild - Moderate - Severe - Very Severe | - Yes - No | - Non-pharmacological - Pharmacological - Patient should speak to health care provider about symptoms at next clinic visit - Patient should seek immediate assistance (ER) |
| **Pain** | - None - Rarely - Occasionally - Frequently - Almost Constantly | - Mild - Moderate - Severe - Very Severe | - Yes - No | - Non-pharmacological - Pharmacological - Patient should speak to health care provider about symptoms at next clinic visit - Patient should seek immediate assistance (ER) |
| **Aching Joints (such as elbows, knees and shoulders) or Aching Muscles** | - None - Rarely - Occasionally - Frequently - Almost Constantly | - Mild - Moderate - Severe - Very Severe | - Yes - No | - Non-pharmacological - Pharmacological - Patient should speak to health care provider about symptoms at next clinic visit - Patient should seek immediate assistance (ER) |
| **Shivering or Shaking Chills** | - None - Rarely - Occasionally - Frequently - Almost Constantly | - Mild - Moderate - Severe - Very Severe | - Yes - No | - Non-pharmacological - Pharmacological - Patient should speak to health care provider about symptoms at next clinic visit - Patient should seek immediate assistance (ER) |
| **Chemotherapy Toxicity** | **Frequency** | **Severity** | **Were Recommendations Made?** | **Recommendations Made** |
| **Fatigue, Tiredness or Lack of Energy** | - None - Rarely - Occasionally - Frequently - Almost Constantly | - Mild - Moderate - Severe - Very Severe | - Yes - No | - Non-pharmacological - Pharmacological - Patient should speak to health care provider about symptoms at next clinic visit - Patient should seek immediate assistance (ER) |
| **Additional Symptom: _______________**  **_______________** | - None - Rarely - Occasionally - Frequently - Almost Constantly | - Mild - Moderate - Severe - Very Severe | - Yes - No | - Non-pharmacological - Pharmacological - Patient should speak to health care provider about symptoms at next clinic visit - Patient should seek immediate assistance (ER) |
| **Additional Symptom: _______________**  **_______________** | - None - Rarely - Occasionally - Frequently - Almost Constantly | - Mild - Moderate - Severe - Very Severe | - Yes - No | - Non-pharmacological - Pharmacological - Patient should speak to health care provider about symptoms at next clinic visit - Patient should seek immediate assistance (ER) |
| **Additional Symptom: _______________**  **_______________** | - None - Rarely - Occasionally - Frequently - Almost Constantly | - Mild - Moderate - Severe - Very Severe | - Yes - No | - Non-pharmacological - Pharmacological - Patient should speak to health care provider about symptoms at next clinic visit - Patient should seek immediate assistance (ER) |

**Notes:** ______________________________________________________________________________________________________________________________________________________________________________________________________________________________________________________________________

**Signature:** ______________________________________________ **Date:** ______________________________________________________
